# The relationship between epicardial fat tissue thickness and visceral adipose tissue in lean patients with polycystic ovary syndrome

**DOI:** 10.1186/s13048-015-0197-4

**Published:** 2015-11-06

**Authors:** Dilek Arpaci, Aysel Gurkan Tocoglu, Sabiye Yilmaz, Hasan Ergenc, Ali Tamer, Nurgul Keser, Huseyin Gunduz

**Affiliations:** Division of Endocrinology and Metabolism, Department of Internal Medicine, Faculty of Medicine, Bulent Ecevit University, Zonguldak, Turkey; Department of Internal Medicine, Faculty of Medicine, Sakarya University Training and Research Hospital, Sakarya, Turkey; Department of Cardiology, Sakarya University Training and Research Hospital, Sakarya, Turkey

**Keywords:** Polycystic, Ovary, Epicardial, Adipose

## Abstract

**Background:**

Polycystic ovary syndrome (PCOS) is related to metabolic syndrome, insulin resistance, and cardiovascular metabolic syndromes. This is particularly true for individuals with central and abdominal obesity because visceral abdominal adipose tissue (VAAT) and epicardial adipose tissue (EAT) produce a large number of proinflammatory and proatherogenic cytokines. The present study aimed to determine whether there are changes in VAAT and EAT levels which were considered as indirect predictors for subclinical atherosclerosis in lean patients with PCOS.

**Methods:**

The clinical and demographic characteristics of 35 patients with PCOS and 38 healthy control subjects were recorded for the present study. Additionally, the serum levels of various biochemical parameters were measured and EAT levels were assessed using 2D-transthoracic echocardiography.

**Results:**

There were no significant differences in mean age (*p* = 0.056) or mean body mass index (BMI) (*p* = 0.446) between the patient and control groups. However, the body fat percentage, waist-to-hip ratio, amount of abdominal subcutaneous adipose tissue, and VAAT thickness were higher in the PCOS patient group than in the control group. The amounts of EAT in the patient and control groups were similar (*p* = 0.384). EAT was correlated with BMI, fat mass, waist circumference, and hip circumference but not with any biochemical metabolic parameters including the homeostasis model assessment of insulin resistance index or the levels of triglycerides, low-density lipoprotein cholesterol, and high-density lipoprotein (HDL) cholesterol. However, there was a small positive correlation between the amounts of VAAT and EAT. VAAT was directly correlated with body fat parameters such as BMI, fat mass, and abdominal subcutaneous adipose thickness and inversely correlated with the HDL cholesterol level.

**Conclusions:**

The present study found that increased abdominal adipose tissue in patients with PCOS was associated with atherosclerosis. Additionally, EAT may aid in the determination of the risk of atherosclerosis in patients with PCOS because it is easily measured.

## Background

Polycystic ovary syndrome (PCOS) is a heterogeneous disease that affects 5 to 10 % of women in the reproductive period [1]. Many studies have shown that PCOS is associated with various cardiovascular risk factors such as obesity, insulin resistance, hyperlipidemia, metabolic syndrome, and hypertension [1–3]. Additionally, patients with PCOS have a high incidence of central and abdominal obesity and marked increases in the waist circumference (WC) and waist-to-hip ratio (WHR) [4, 5].

Visceral abdominal adipose tissue (VAAT) surrounds the internal organs, and increased amounts of VAAT are more important than increased levels of subcutaneous fat in terms of the risks of metabolic syndrome, insulin resistance, and cardiovascular mortality [6]. Epicardial adipose tissue (EAT) and visceral adipose tissue (VAT), located between the myocardium and visceral epicardium, respectively, are derived from the same origin [7]. This is important because both of these body fat tissues produce large numbers of proinflammatory and proatherogenic cytokines [8, 9]. The reported findings regarding abdominal fat tissue and EAT in patients with PCOS are controversial [8–21]. For example, patients with PCOS have been shown to have increased [10–14], similar [15–17], or decreased [18] amounts of abdominal fat. Similarly, the amount of EAT in patients with PCOS has been reported to be increased and unchanged compared with healthy control groups [19–21]. However, not all patients with PCOS are obese; in fact, a 2001 study of 346 patients with PCOS found that 56 % of such patients are lean [22]; 56.0 % had a body mass index (BMI) of < 25 kg/m^2^, 11.3 % had a BMI of 25 to 27 kg/m^2^, and 32.7 % had a BMI of ≥ 27 kg/m^2^ [22]. Thus, the present study aimed to determine whether there are changes in the amounts of VAAT and EAT in lean patients with PCOS compared with healthy control subjects.

## Methods

### Selection of subjects

The present study included 38 healthy control subjects and 35 patients with PCOS and concurrent hyperandrogenism and/or ovulatory dysfunction who were admitted to the Endocrinology and Metabolism Department of Sakarya Training and Research Hospital at Sakarya University from January 2013 to June 2014. Some of these patients were diagnosed by a gynecologist, and some were diagnosed by the present authors based on the presence of two of the three criteria from the Rotterdam European Society for Human Reproduction and Embryology/American Society for Reproductive Medicine (ESHRE/ASRM) for PCOS: a) oligomenorrhea, amenorrhea, or anovulation; b) the presence of clinical or biochemical hyperandrogenism; and/or c) the presence of polycystic ovaries as determined by a pelvic ultrasound [22]. The control group comprised healthy secretaries, nurses, and doctors from our hospital who volunteered for the study and had regular menstrual cycles, normal androgen levels, the absence of hirsutism, and no polycystic ovary as determined by a pelvic ultrasound. The demographic data of the patient and control groups were recorded. The present study was approved by the Sakarya University Faculty of Medicine Ethics Committee (Date: 24.02.2014; No. 27), and all participants provided written informed consent.

### Exclusion criteria

Subjects were excluded from the present study if they had history of smoking, diabetes, or hypertension; had been diagnosed with Cushing’s syndrome (based on the 1-mg dexamethasone suppression test) or non-classic congenital adrenal hyperplasia (based on a 17-OH progesterone level of > 10 ng/dL after stimulation); exhibited cardiac disease; and/or had used antidiabetic, antihypertensive, antilipidemic, or oral contraceptive drugs within the past 3 months.

### Measurements

The height and weight of all subjects were measured, and their BMI was calculated as the weight in kilograms divided by the square of height in meters. WC was measured from the narrowest part of the body between the iliac crest and the rib, and hip circumference (HC) was measured at the widest part of the hips. The WHR was calculated as the ratio of WC to HC [21].

### Body composition analysis

The basal metabolic rate, body fat percentage, and total body water of each patient were evaluated with a Tanita Body Composition Analyzer (Model TBF-300; Tanita Corporation, Itabashi-ku, Tokyo, Japan) while the patient was in a standing position without shoes and with light clothing on after a ≥ 8-h fast with sufficient hydration. Abdominal subcutaneous fat tissue thickness and visceral abdominal fat tissue thickness were recorded using bioelectrical impedance with the Tanita Abdominal Fat Analyzer (AB-140 Viscan; Tanita Corporation, Tokyo, Japan). The blood pressure of each patient was assessed after at least 10 min of rest with a sphygmomanometer (ERKA; Bad Tölz, Germany); two measurements were performed, and the average of the blood pressure measurements was calculated [23].

### Biochemical analysis

Blood samples were obtained from the patients in the morning after at least 8 h of fasting. The fasting plasma glucose (FPG) and fasting insulin levels of the patients were measured, and the homeostatic model assessment-insulin resistance (HOMA-IR) index was calculated using the following formula: (FPG [mg/dL] × fasting plasma insulin [μIU/mL] / 405). If the HOMA-IR index was > 2.7, insulin resistance was considered to be present [24]. Serum lipid levels of low-density lipoprotein (LDL) cholesterol, high-density lipoprotein (HDL) cholesterol, and triglycerides (TG) were measured using xylidine blue with an end-point colorimetric method (Roche Diagnostics GmbH; Mannheim, Germany). FPG levels were measured with a hexokinase method (Roche Diagnostics GmbH).

### Echocardiography

All patients were directed to the Department of Cardiology at Sakarya Training and Research Hospital, and the EAT thickness was measured using 2D-transthoracic echocardiography by the same cardiologist. The parasternal long- and short-axis images of EAT, which allow for the most accurate measurement from the right ventricle, were obtained using a standard parasternal image in the left lateral decubitus position. EAT was defined as the echo-free space between the outer wall of the myocardium and the visceral layer of pericardium at end-systole in the right ventricle [25].

### Statistical analysis

All statistical analyses were performed with SPSS software, version 15 (SPSS, Inc., Chicago, IL, USA). Nonparametric tests were utilized due to the prevalence of variables. The Mann–Whitney *U* test was applied to assess differences between groups, and Spearman’s test was applied to assess correlations between the variables. A *p* value of < 0.05 was considered to indicate statistical significance, and a correlation was considered to be present if Spearman’s value was ≥ 0.50. Continuous variables are expressed as either the mean ± standard deviation (SD) or the median (minimum–maximum), and categorical variables are expressed as either frequency or percentage. Continuous variables were compared with an independent-samples t-tests or the Mann–Whitney *U* test, and categorical variables were compared using Pearson’s chi-square test. A *p* value of < 0.05 was considered to indicate statistical significance for all tests.

## Results

The body fat distribution, total body water, WC, HC, WHR, amount of abdominal subcutaneous adipose tissue, VAAT thickness, blood pressure, and levels of LDL cholesterol, HDL cholesterol, are TG are provided in Table 1. There were no significant differences in the mean age (*p* = 0.056) or mean BMI (*p* = 0.446) between the patient and control groups, but the body fat percentage, WHR, amount of abdominal subcutaneous adipose tissue, and VAAT thickness were higher in the patient group. However, the patient and control groups had similar amounts of EAT (*p* = 0.384) (Table 1).

**Table 1:** Comparison of demographic, body analysis and laboratory parameters of the patient and the control group

Feature	Patients (*n* = 35)	Controls (*n* = 38)	*P* value
Age (years)	25.16 ± 4.12	27.44 ± 4.31	0.056
BMI (kg/m^2^)	25.60 ± 5.22	23.67 ± 3.70	0.404
Fat mass (%)	31.74 ± 10.12	26.33 ± 7.61	**0.042**
Total body water (kg)	34.11 ± 4.48	32.72 ± 2.33	0.193
Waist circumference (cm)	91.76 ± 18.95	86.50 ± 9.71	0.677
Hip circumference (cm)	103.56 ± 15.27	100.55 ± 7.48	0.557
WHR	0.94 ± 0.06	0.89 ± 0.04	**0.007**
VAAT thickness	9.24 ± 4.67	6.77 ± 2.68	**0.042**
Abdominal subcutaneous adipose tissue thickness	40.48 ± 8.83	32.86 ± 9.48	**0.008**
Systolic blood pressure (mmHg)	120.10 ± 7.78	120.28 ± 7.28	0.979
Diastolic blood pressure (mmHg)	70.26 ± 7.40	72.76 ± 9.21	0.318
FPG (mg/dL)	89.16 ± 9.12	87.72 ± 7.92	0.565
Insulin (μIU/ mL)	9.77 ± 6.27	6.80 ± 3.45	0.073
HOMA-IR	2.13 ± 1.33	1.60 ± 0.67	0.082
TG (mg/dL)	11.52 ± 53.81	85.52 ± 31.49	0.058
LDL (mg/dL)	106.40 ± 35.57	96.38 ± 28.73	0.305
HDL (mg/dL)	55.69 ± 16.25	58.90 ± 11.96	0.300
EAT (mm)	4.72 ± 0.88	4.43 ± 1.31	0.384

*BMI* body mass index, *VAAT* visceral abdominal adipose tissue, *FPG* fasting plasma glucose, *HOMA-IR* Homeostatic Model Assessment-insulin resistance, *TG* triglyceride, *LDL* low density lipoprotein, *HDL* high density lipoprotein, *EAT* epicardial adipose tissue. Continuous variables were compared with an independent-samples t-tests or the Mann–Whitney *U* test, and categorical variables were compared using Pearson’s chi-square test. A *p* value of <0.05 was considered to indicate statistical significance for all tests

EAT had a significantly positive correlation with BMI (r = 0.260, *p* = 0.034), fat mass (r = 0.250, *p* = 0.041), WC (r = 0.301, *p* = 0.016), and abdominal circumference (r = 0.254, *p* = 0.043), but it was not correlated with the HOMA-IR index or the levels of TG, LDL cholesterol, or HDL cholesterol (*p* > 0.05) (Table 2). There was a small positive correlation between VAAT and EAT (r = 0.248, *p* = 0.048). VAAT was also directly associated with BMI (r = 0.921, *p* < 0.01), fat mass (r = 0.941, *p* < 0.01), WC (r = 0.941, *p* < 0.01), HC (r = 0.876, *p* < 0.01), abdominal subcutaneous adipose thickness (r = 0.896, *p* < 0.01), the HOMA-IR index (r = 0.618, *p* < 0.01), and the levels of TG (r = 0.388, *p* < 0.01) and LDL cholesterol (r = 0.288, *p* = 0.016). Conversely, VAAT was inversely associated with the HDL cholesterol level (r = −0.488, *p* < 0.01) (Table 3).

**Table 2:** Correlation analysis between EAT and variables

Variable	r value	*p* value
BMI (kg/m^2^)	0.260	**0.034**
Fat mass (%)	0.250	**0.041**
Waist circumference (cm)	0.301	**0.016**
Hip circumference (cm)	0.254	**0.043**
HOMA-IR	0.119	0.490
TG (mg/dL)	0.076	0.550
LDL (mg/dL)	0.158	0.209
HDL (mg/dL)	−0.185	0.141

*BMI* body mass index, *HOMA-IR* Homeostatic Model Assessment-insulin resistance, *TG* triglyceride, *LDL* low density lipoprotein, *HDL* high density lipoprotein, *EAT* epicardial adipose tissue. Continuous variables were compared with an independent-samples t-tests or the Mann–Whitney *U* test, and categorical variables were compared using Pearson’s chi-square test. A *p* value of <0.05 was considered to indicate statistical significance for all tests

**Table 3:** Correlation analysis between VAAT and variables

Variable	r value	*P* value
EAT (mm)	0.248	**0.048**
BMI (kg/m^2^)	0.921	<0.01
Fat mass (%)	0.941	<0.01
Waist circumference (cm)	0.941	<0.01
Hip circumference (cm)	0.876	<0.01
Abdominal subcutaneous adipose tissue thickness	0.896	<0.01
HOMA-IR	0.618	<0.01
TG (mg/dL)	0.388	<0.01
LDL (mg/dL)	0.288	**0.016**
HDL (mg/dL)	−0.488	<0.01

*BMI* body mass index, *HOMA-IR* Homeostatic Model Assessment-insulin resistance, *TG* triglyceride, *LDL* low density lipoprotein, *HDL* high density lipoprotein, *EAT* epicardial adipose tissue. Continuous variables were compared with an independent-samples t-tests or the Mann–Whitney *U* test, and categorical variables were compared using Pearson’s chi-square test. A *p* value of <0.05 was considered to indicate statistical significance for all tests

## Discussion

Although the patient and control groups in the present study had similar ages and BMIs, the lean patients with PCOS exhibited a higher WHR, VAAT, and abdominal subcutaneous fat tissue thickness than did the control group. In contrast, there were no significant differences in EAT. However, EAT was significantly correlated with VAAT, BMI, fat mass, WC, and HC.

Previous studies have shown a positive correlation between EAT thickness and VAAT thickness [26–28] independent of obesity [26, 27], and this correlation seems to be more important than WC [28]. Although there is an increased amount of EAT in obese patients with than without PCOS [19–21], EAT is not different between lean patients with PCOS and the normal population [21, 29]. Similarly, the present study found no differences in EAT between the two groups. Compared with normal individuals, patients with PCOS exhibit increases in total fat mass and organ-specific VAT [29]. Furthermore, these increases are positively correlated with both systolic and diastolic blood pressure and the levels of fasting glucose, insulin, LDL cholesterol, TG, and transaminases but negatively correlated with the insulin sensitivity index and HDL cholesterol. Likewise, the present study found that fat mass, abdominal subcutaneous adipose tissue, and VAAT thickness were higher in the patient group than in the control group. Additionally, VAAT thickness was positively correlated with BMI, fat mass, WC, HC, abdominal subcutaneous adipose tissue thickness, and the levels of TG and LDL cholesterol but negatively correlated with HDL cholesterol.

Sahin et al. [21] reported that EAT is positively correlated with age, BMI, WC, the glucose level, the HOMA-IR index, and the TG level. Similarly, the present study found that EAT was positively correlated with BMI, WC, HC, and VAAT; however, in contrast to those previous findings, EAT was not correlated with fasting glucose, the HOMA-IR index, or the lipid parameters. This may be due to the fact that the patients with PCOS in the present study were lean rather than obese. Another study found that EAT is correlated with BMI, WC, VAT thickness, and insulin resistance [30]. In the present study, EAT was positively correlated with BMI, WC, and VAAT but not with the HOMA-IR index. EAT is reportedly more closely associated with VAT than with total body fat [28, 30].

In studies employing magnetic resonance imaging (MRI), the abdominal adipose tissue thicknesses of patients with PCOS and normal control subjects did not significantly differ [16, 31–33]. It has also been shown that VAAT thickness is increased only in mildly obese patients with PCOS relative to control subjects [15] and that obesity predicts insulin resistance independently of PCOS [31]. Furthermore, a study conducted using a bioimpedance device found that there was less VAT in lean patients with PCOS than in the control group [18]. In contrast, the present study found greater VAAT thickness in lean patients with PCOS than in the control group despite the fact that the EAT thickness did not change.

The gold standard tests for measuring VAT are MRI and computed tomography (CT) [34]. Thus, a limitation of the present study may be that the amounts of abdominal subcutaneous adipose tissue and VAT were assessed using bioelectrical impedance. However, it is difficult to measure adipose tissue using CT or MRI because of cost-effectiveness, the application of radiation, and the use of contrast media. Additionally, previous studies have shown that the results of bioelectrical impedance tests and CT scans when measuring VAT are closely correlated [35]. Consequently, given that the present study found a small positive correlation between EAT thickness and VAAT thickness, echocardiography would appear to be an easy, simple, noninvasive, reliable, and accessible method for the measurement of these parameters relative to the use of MRI scans [25]. EAT is important for the determination of both VAAT thickness and cardiovascular risk [26, 36], and increased abdominal adipose tissue is related to an increased risk of atherosclerosis [37] and mortality [38]. Because it can be difficult to measure abdominal adipose tissue, it may be more economical and efficient to determine these risk factors by measuring EAT.

## Conclusions

The present study observed several associations between EAT thickness and cardiovascular risk in patients with PCOS. Because of the difficulties related to the measurement of abdominal adipose tissue thickness, the assessment of EAT may be a relatively easy-to-use but important tool for the determination of cardiovascular risk.

## Abbreviations

PCOS: Polycystic ovary syndrome
VAAT: Visceral abdominal adipose tissue
EAT: Epicardial adipose tissue
BMI: Body mass index
